# Standard Echocardiographic View Recognition in Diagnosis of Congenital Heart Defects in Children Using Deep Learning Based on Knowledge Distillation

**DOI:** 10.3389/fped.2021.770182

**Published:** 2022-01-18

**Authors:** Lanping Wu, Bin Dong, Xiaoqing Liu, Wenjing Hong, Lijun Chen, Kunlun Gao, Qiuyang Sheng, Yizhou Yu, Liebin Zhao, Yuqi Zhang

**Affiliations:** ^1^Department of Pediatric Cardiology, Shanghai Children's Medical Center, School of Medicine, Shanghai Jiao Tong University, Shanghai, China; ^2^Shanghai Engineering Research Center of Intelligence Pediatrics, Shanghai, China; ^3^Deepwise Artificial Intelligence Laboratory, Beijing, China

**Keywords:** standard echocardiographic view, congenital heart defect, deep learning, convolutional neural network, knowledge distillation

## Abstract

Standard echocardiographic view recognition is a prerequisite for automatic diagnosis of congenital heart defects (CHDs). This study aims to evaluate the feasibility and accuracy of standard echocardiographic view recognition in the diagnosis of CHDs in children using convolutional neural networks (CNNs). A new deep learning-based neural network method was proposed to automatically and efficiently identify commonly used standard echocardiographic views. A total of 367,571 echocardiographic image slices from 3,772 subjects were used to train and validate the proposed echocardiographic view recognition model where 23 standard echocardiographic views commonly used to diagnose CHDs in children were identified. The F1 scores of a majority of views were all ≥0.90, including subcostal sagittal/coronal view of the atrium septum, apical four-chamber view, apical five-chamber view, low parasternal four-chamber view, sax-mid, sax-basal, parasternal long-axis view of the left ventricle (PSLV), suprasternal long-axis view of the entire aortic arch, M-mode echocardiographic recording of the aortic (M-AO) and the left ventricle at the level of the papillary muscle (M-LV), Doppler recording from the mitral valve (DP-MV), the tricuspid valve (DP-TV), the ascending aorta (DP-AAO), the pulmonary valve (DP-PV), and the descending aorta (DP-DAO). This study provides a solid foundation for the subsequent use of artificial intelligence (AI) to identify CHDs in children.

## Introduction

Congenital heart defects (CHDs) are the most common birth defects in China, with an incidence rate of approximately 0.9% in live births, and are the main causes of death in children aged 0–5 years (1). Precise preoperative diagnosis helps to develop the most reasonable surgery and interventional treatment plan for CHDs. Transthoracic echocardiography (TTE) can provide sufficient information about the structure of the heart to evaluate hemodynamics and cardiac function. It is currently the most commonly used non-invasive examination method for CHDs. Many factors contribute to the heterogeneity of echocardiographic diagnosis, such as inherent pulse variability in the heart, speckled noise and artifacts in echocardiogram, and inter- and intra-class differences in standard echocardiographic views (2). The anatomical structure and spatial configuration of CHDs are complex and changeable, and accurate diagnosis through TTE is complicated and time-consuming, and it is heavily dependent on the accurate judgment of each echocardiographic view by experienced cardiologists. In China, there is a lack of experienced cardiologists at the grassroots level, especially in rural areas. It takes a great effort to train an experienced doctor. In addition, many cardiologists lack experience in diagnosing CHDs. Experienced cardiac surgery centers usually have long waiting lists for appointments, and cardiologists are overworked, leading to an imbalance between supply and demand. Therefore, it is very necessary to establish an automatic diagnosis system of CHDs to alleviate the difficulty of CHD diagnosis at the grassroots level.

In recent years, artificial intelligence (AI) based image recognition technology, especially deep learning via convolutional neural networks (CNNs), have been greatly improved and increasingly applied to diagnostic imaging in medical fields (3–11). Currently, AI based analytic software has been applied to the research of valvular diseases. Jin et al. (12) has compared the accuracy of anatomical intelligence ultrasound (AIUS) and manual depiction of the mitral valve prolapse degree and found that AIUS can significantly improve accuracy and reduce analysis time. According to the research of Choi et al. (13), the aortic valve (AV) regurgitation volume measured by three-dimensional full volume color Doppler echocardiography has a high correlation and consistency with those measured by magnetic resonance spectrum, which can be used to quantitatively evaluate the AV disease.

Standard echocardiographic view recognition provides the foundation for the clinical diagnosis of heart diseases. Recent CNNs based on deep learning methods have been applied in standard echocardiographic view recognition and achieved superior performance than traditional methods. Extensive research has been focused on fetal ultrasound (14, 15). Baumgartner et al. (14) proposed a two-dimensional full CNN containing six convolutional modules, which replaced the commonly used full connection layer with the convolutional and global average pooling layers, and recognized 12 standard views of fetal ultrasound with an average accuracy of 69% and average recall rate of 80%. Sridar et al. (15) also started with fetal ultrasound data and used the AlexNet neural network pre-trained with a natural image dataset to identify 14 views. They designed a strategy for simultaneous training using two parallel networks. The input of one network was the entire ultrasonic image used to learn the global semantic information of the image. The input of the other network was random local segmentation of ultrasonic images used to learn the local features of the image. The average accuracy rate and average recall rate were 76.47 and 75.41%, respectively. Zhang et al. (16) attempted to develop a fully automated echocardiographic clinical diagnosis system consisting of standard view recognition, cardiac structure segmentation, and heart disease diagnosis. They applied a 13-layer CNN network to standard view recognition, and obtained a global accuracy of 84% on standard view recognition using 277 cases through a five-fold cross validation. Their model can be used to detect hypertrophic cardiomyopathy, pulmonary arterial hypertension, and cardiac amyloidosis. However, their work was far from the diagnosis of common CHDs since the standard views they used were mainly derived from five views including parasternal long axis (PSLA), parasternal short axis, apical 2-chamber (A2C), apical 3-chamber (A3C), apical 4-chamber (A4C).

In this study, we used 367,571 echocardiographic images from 3,772 subjects to build a 24-category classification model that can identify 23 standard echocardiographic views and one “other” view. To our knowledge, this is the first work that covers the most commonly used CHD diagnostic views. As we all know, there is a trade-off between the complexity and accuracy of the model. Achieving high accuracy and timeliness simultaneously is challenging, especially when a large number of views need to be identified. Our experimental results demonstrate that the proposed knowledge distillation method maximizes the accuracy of the network while keeping the complexity of the network unchanged and has important clinical significance. Ultrasound examination of CHDs is a dynamic scanning process and images collected are mostly non-standard views and unevenly distributed. Compared with existing work, the data set used in our study is the largest taken from a real historical examination database, closer to the routine CHD diagnosis, which is another clinical contribution of this work.

## Methods

### Subjects

The experimental data consists of a training data set (3,409 cases and 247,750 TTE images) and a test data set (363 cases and 119,821 TTE images), both from the Shanghai Children's Medical Center. The TTE images are retrieved from a historical real examination database in PACS system (Table 1). The 363 cases of the test data set include 193 male cases and 170 female cases, with an average age of 5.6 years. There were 170 healthy children, 102 children with CHDs, and 91 children with CHDs after surgery. Table 2 lists the characteristics of the test dataset. The machines used were mainly Philips iE33 and EPIC 7C. Six professional ultrasound doctors participated in the data annotation, of which four ultrasound doctors participated in the annotation of the training data set, and two ultrasound doctors participated in the annotation of the test data set. To avoid human bias, all images were annotated and confirmed by two doctors. More specifically, each image was annotated by a senior resident or attending physician with more than 5 years of experience, and then reviewed and confirmed by an associate chief physician or chief physician with at least 10 years of experience. All data was tailored to anonymize patient information.

**Table 1:** View distribution of our data.

**Views**	**Training data set**	**Test data set**
sub4C	2,679	3,042
subSALV	2,698	1,143
subSAS	26,183	11,922
subCAS	11,552	8,271
subRVOT	12,094	5,458
A4C	23,845	14,313
A5C	5,034	2,383
LPS4C	20,082	14,414
LPS5C	12,481	4,657
sax-basal	17,368	10,459
sax-mid	6,393	5,813
PSLV	16,610	9,280
PSPA	18,510	7,419
supAO	7,684	5,070
DP-MV	1,445	586
DP-TV	1,414	519
DP-AAO	1,500	665
DP-PV	1,497	769
DP-DAO	857	449
DP-OTHER	2,186	1,418
M-AO	600	389
M-LV	1,030	220
M-OTHER	34	20
Others	53,974	11,142
Total	247,750	119,821

**Table 2:** Characteristics comparison between the CHD group and the normal groups of the test data set.

	**CHD group (*n* = 193)**	**Normal group** **(*n* = 170)**	***P*-value**	**Statistical method**
Age (years)	4.42 (1.33–8.17)	4.67 (2.56–7.52)	0.5028	Mann Whitney test
Female/Male	103/90	91/79	0.9754	Chi-square test
Associated cardiac conditions	ASD (*n* = 29) PDA (*n* = 9) VSD (*n* = 30) PS (*n* = 9) AS (*N* = 4) TOF/DORV (*n* = 4) Other CHD (*n* = 17) CHD after operation (*n* = 91)	KD (*n* = 23) Normal (*n* = 147)		

*ASD, atrial septal defect; VSD, ventricular septal defect; PDA, patent ductus arteriosus; PS, pulmonary stenosis; AS, aortic stenosis; TOF, tetralogy of fallot; DORV, double outlet of right ventricle; Age, Median (25% Percentile−75% Percentile)*.

### Data Preprocessing

Usually, the boundary area of each frame of echocardiogram contains a large amount of background information that is not useful for diagnosis. In order to extract the discriminative features embedded in the image, the central fan-shaped area was cropped, and each cropped image frame was resized to a fixed size of 192 × 256 pixels.

### Standard Echocardiographic View Recognition

In clinical practice, the diagnosis of various CHDs involves a series of standard scans. Standard echocardiographic view recognition is a prerequisite for automatic CHD diagnosis. In this study, we established a 24-category classification model that can recognize 23 standard echocardiographic views commonly used to diagnose CHDs in children and one “others.” As shown in Table 1, the 23 views include subcostal four-chamber view (sub4C), subcostal short-axis view of the left ventricle (subSALV), subcostal sagittal view of the atrium septum (subSAS), subcostal coronal view of the atrium septum (subCAS), subcostal short-axis view through the right ventricular outflow tract (subRVOT), apical four-chamber view (A4C), apical five-chamber view (A5C), low parasternal four-chamber view (LPS4C), low parasternal five-chamber view (LPS5C), parasternal short-axis view at the base of the heart (basal short axis, sax-basal), parasternal short-axis view at the level of the mitral valve (short axis at mid, sax-mid), parasternal long-axis view of the left ventricle (PSLV), parasternal view of the pulmonary artery (PSPA), suprasternal long-axis view of the entire aortic arch (supAO), Doppler recording from the mitral valve (DP-MV), the tricuspid valve (DP-TV), the ascending aorta (DP-AAO), the pulmonary valve (DP-PV), the descending aorta (DP-DAO), other Doppler recordings (DP-OTHER), and M-mode echocardiographic recording of the aortic (M-AO), the left ventricle at the level of the papillary muscle (M-LV), other M-mode echocardiographic recordings (M-OTHER). Except for these 23 views, all other views are classified as “others.”

**Figure 1:**
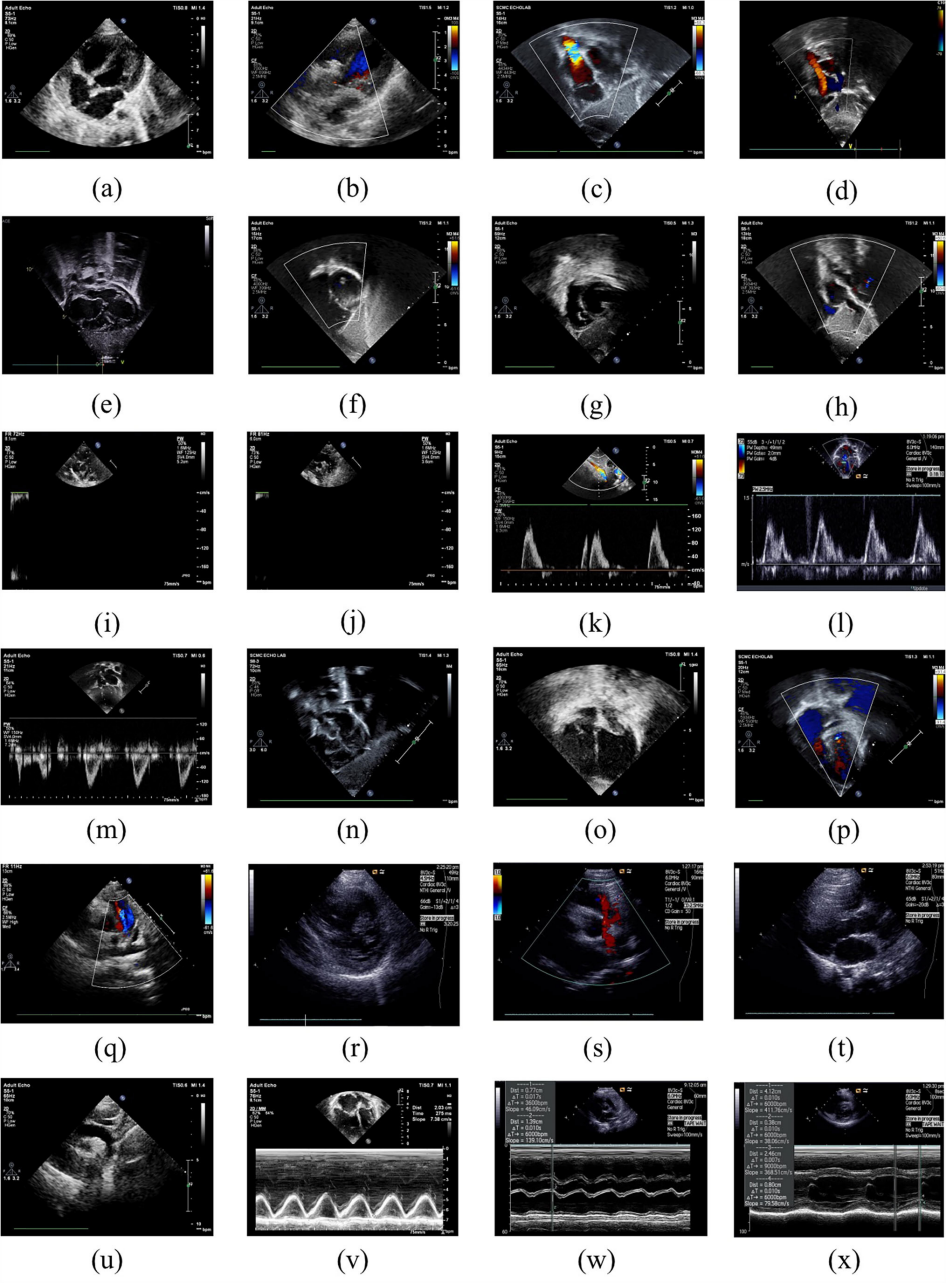
Example images of the 24 standard echocardiographic views: **(a)** LPS4C; **(b)** LPS5C; **(c)** subCAS; **(d)** subSAS; **(e)** sub4C; **(f)** subRVOT; **(g)** subSALV; **(h)** DP-MV; **(i)** DP-PV; **(j)** DP-DAO; **(k)** DP-OTHER; **(l)** DP-TV; **(m)** DP-AAO; **(n)** OTHER; **(o)** A4C; **(p)** A5C; **(q)** sax-basal; **(r)** sax-mid; **(s)** PSLA; **(t)** PSPA; **(u)** supAO; **(v)** M-OTHER; **(w)** M-AO; **(x)** M-LV.

Figure 1 demonstrated the example images of the 24 standard echocardiographic views. Among these views, subcostal views are of great significance for the diagnosis of congenital heart disease in infants and young children, especially for the diagnosis of atrial septal defect (ASD). Apical and low parasternal views are mainly used for the diagnosis of ASD, ventricular septal defect (VSD), tetralogy of Fallot (TOF), and atrioventricular valve diseases. The parasternal views are mainly used for the diagnosis of AV, pulmonary valve (PV) diseases, ASD, VSD, TOF, and patent ductus arteriosus (PDA). M-mode ultrasound is mainly measured next to the parasternal to assess the size of the heart chamber and the systolic function of the left ventricle. Pulse Doppler is mainly used to measure the blood flow velocity of various valves and blood vessels to determine whether there is stenosis.

**Figure 2:**
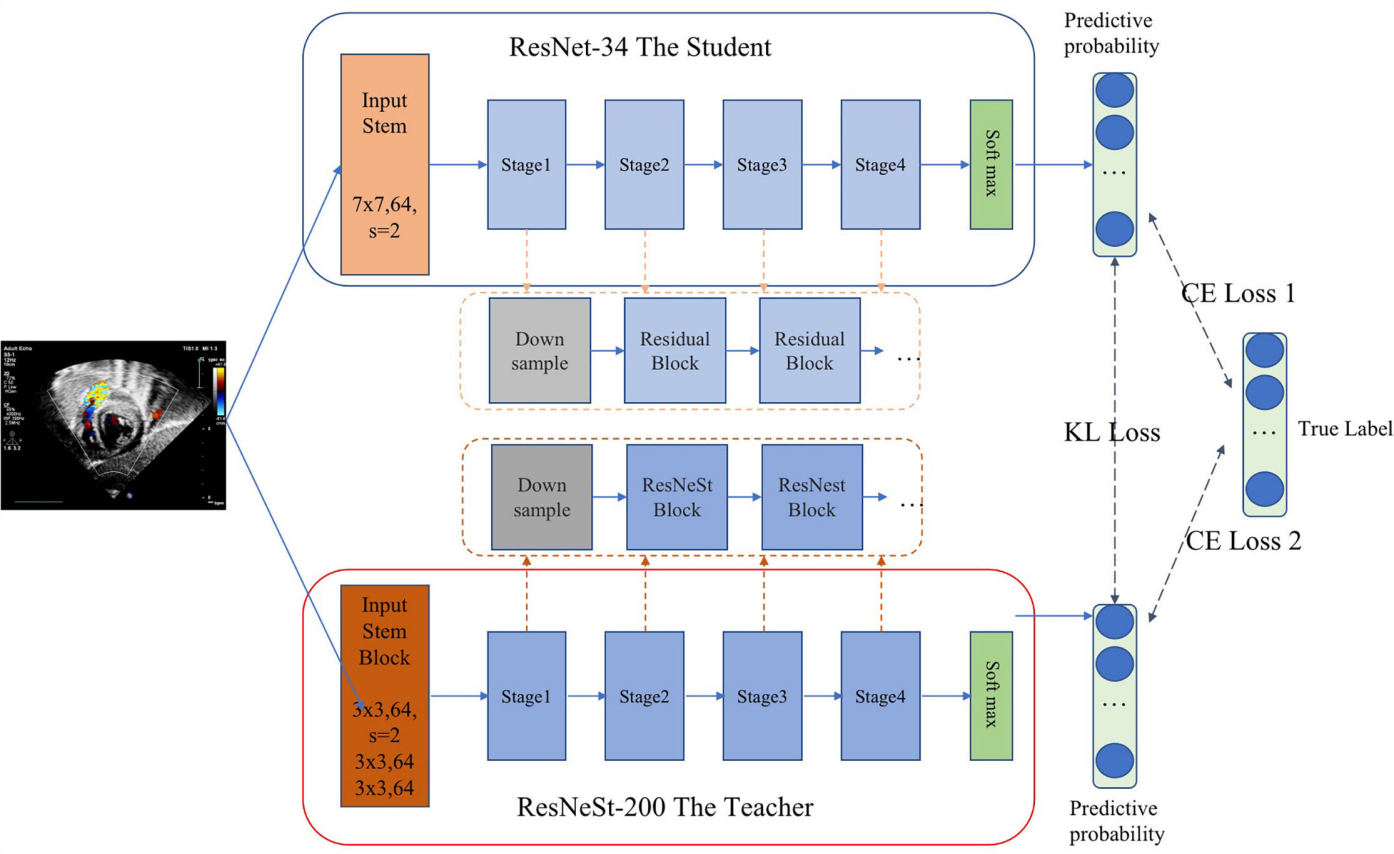
The proposed network architecture for standard echocardiographic view recognition.

As we all know, there is a trade-off between the complexity and accuracy of the model. Achieving high accuracy and timeliness simultaneously is challenging, especially when a large number of views need to be identified. Inspired by the idea of probabilistic knowledge transfer method (17), which considered knowledge distillation as a metric learning problem, we proposed a CNN method that performed standard echocardiographic view recognition through knowledge distillation. Knowledge distillation enables the student network learn the generalization ability of the teacher network, through replacing the hard original one-hot label with the soft label, and learn the ability to distinguish similar features (17–20). Therefore, on the one hand, the knowledge distillation method compresses the model, on the other hand, it enhances the generalization ability of the model. As shown in Figure 2, ResNet-34 (21) was used as the student model and ResNeSt-200 (22) was applied as the teacher model. Our distillation method works by matching the probability distribution of logits of the student and the teacher. In each iteration of the training phase, the student learn from data by minimizing the cross entropy loss. And meanwhile with Kullback-Leibler divergence as the loss function, the output logits of the student will be constantly close to that of the teacher. As a result, we balanced the performance of the model and the complexity of running time through realizing the knowledge transfer from the teacher model to the student model.

### Model Training

We trained the teacher model and the student model simultaneously and also applied transfer learning to the pre-trained backbone network using the large-scale natural image dataset ImageNet (23). To enhance the diversity and robustness of data, a series of online data augmentations including horizontal and/or vertical flipping with a random probability of *p* = 0.5 and random rotation with a random probability of *p* = 0.5 were also implemented. Stochastic gradient descent (SGD) was used as the optimizer with an initial learning rate of 0.001, a decay factor of 0.1 for every 10 epochs, and a mini-batch size of 128.

All codes were implemented using Python 3.7 and Pytorch 1.4.0. The experiments were carried out on a workstation platform with 8 NVIDIA TITAN RTX GPUs, 24GB GPU memory, 256G RAM, and 80 Intel(R) Xeon(R) Gold 6248 CPU @ 2.50GHz, using Ubuntu 16.04.

## Results

In this study, four performance evaluation metrics including *Precision, Sensitivity, Specificity*, and *F1 Score* were applied to validate the performance of the proposed view recognition model and they are defined as follows:


(1) $$Precision\;=\;\frac{TP}{TP\;+\;FP}$$

(2) $$Sensitivity\;=\;Recall\;=\;\frac{TP}{TP\;+\;FN}$$

(3) $$Specificity\;=\;\frac{TN}{TN\;+\;FP}$$

(4) $$F1\;=\;2x\frac{Precision\;*\;Recall}{Precision\;+\;Recall}$$

where *TP, FP, TN*, and *FN* are the true positive, false positive, true negative, and false negative rates, respectively. *TP* and *TN* represent the positives and negatives of correct predictions with respect to the ground truth. *FP* and *FN* represent the positives and negatives of incorrect predictions with respect to the ground truth. *F1 Score* is the harmonic average of precision and recall with a value ranged in [0–1]. The higher the value means that the model has better performance.

Common CHDs in children include ASDs, VSD, PDA, pulmonary stenosis (PS), aortic stenosis (AS), TOF, and so on. Table 3 lists the diagnosable CHDs corresponding to each standard echocardiographic view. More specifically, the most commonly used ASD diagnostic views include sub4C, subSAS, subCAS, LPS4C, and sax-basal. The most commonly used views for the diagnosis of VSD are sub4C, subRVOT, subSALV, A4C, A5C, LPS4C, LPS5C, and PSLA. The most commonly used views for PVS diagnosis include the subRVOT, sax-basal, and PSPA. The most commonly used views for diagnosing PDA include PSPA and supAO. The most commonly used views to diagnose TOF include sub4C, subRVOT, A4C, A5C, LPS4C, LPS5C, sax-basal, PSPA, and PSLA. M-AO and M-LV are used for heart size measurement and heart function calculation. The blood flow spectrum of the four valves and descending aorta in these 23 views provide the basis for the subsequent diagnosis of valvular and aortic arch diseases. As shown in Table 3, the F1 scores of the subCAS+SAS, A4C, A5C, PSLV, LPS4C, sax-mid, sax-basal, supAO, M-AO, M-LV, M-other, DP-MV, DP-TV, DP-AO, and DP-DAO are all higher or close to 0.90.

**Table 3:** Performance evaluation for different views.

**Views**	**Precision** **(95%CI)**	**Sensitivity** **(95%CI)**	**Specificity** **(95%CI)**	**F1 Score** **(95%CI)**	**CHD Type**
subSAS	0.856 (0.849–0.862)	0.846 (0.840–0.852)	0.984 (0.984–0.985)	0.851 (0.851–0.851)	ASD
subCAS	0.659 (0.650–0.668)	0.779 (0.771–0.788)	0.970 (0.969–0.971)	0.714 (0.714–0.714)	ASD
subSAS+CAS	0.890 (0.886–0.894)	0.951 (0.948–0.954)	0.976 (0.975–0.977)	0.919 (0.919–0.919)	ASD
subRVOT	0.828 (0.818–0.838)	0.839 (0.829–0.849)	0.992 (0.991–0.992)	0.833 (0.833–0.833)	VSD, PS, TOF
sub4C	0.682 (0.663–0.700)	0.544 (0.527–0.562)	0.993 (0.993–0.994)	0.605 (0.605–0.605)	ASD, VSD
subSALV	0.666 (0.631–0.701)	0.410 (0.382–0.439)	0.998 (0.998–0.998)	0.508 (0.508–0.508)	VSD
A4C	0.925 (0.921–0.929)	0.972 (0.969–0.974)	0.989 (0.989–0.990)	0.948 (0.948–0.948)	VSD, ASD, CAVC
A5C	0.889 (0.877–0.902)	0.917 (0.906–0.928)	0.998 (0.997–0.998)	0.903 (0.903–0.903)	VSD, TOF
LPS4C	0.901 (0.896–0.906)	0.872 (0.867–0.878)	0.987 (0.986–0.988)	0.886 (0.886–0.886)	ASD, VSD, CAVC
LPS5C	0.802 (0.790–0.813)	0.790 (0.779–0.802)	0.992 (0.992–0.993)	0.796 (0.796–0.796)	VSD, TOF
PSLV	0.954 (0.949–0.958)	0.933 (0.928–0.938)	0.996 (0.996–0.997)	0.943 (0.943–0.943)	VSD, AS, TOF
PSPA	0.855 (0.847–0.863)	0.846 (0.837–0.854)	0.991 (0.990–0.991)	0.850 (0.850–0.850)	VSD, PS, PDA, TOF
sax-mid	0.972 (0.967–0.976)	0.920 (0.913–0.927)	0.999 (0.998–0.999)	0.945 (0.945–0.945)	VSD, CAVC
sax-basal	0.880 (0.874–0.886)	0.871 (0.865–0.878)	0.989 (0.988–0.989)	0.876 (0.876–0.876)	AS, ASD, VSD, PS, TOF
supAO	0.903 (0.895–0.912)	0.891 (0.882–0.900)	0.996 (0.995–0.996)	0.897 (0.897–0.897)	PDA, COA
M-AO	0.959 (0.939–0.979)	0.900 (0.870–0.930)	1.000 (1.000–1.000)	0.928 (0.928–0.928)	Assess the size of the heart chamber and the systolic function of the left ventricle
M-LV	0.850 (0.807–0.894)	0.982 (0.964–0.999)	1.000 (1.000–1.000)	0.911 (0.911–0.912)	
M-OTHER	0.944 (0.839–1.000)	0.850 (0.694–1.000)	1.000 (1.000–1.000)	0.895 (0.895–0.895)	
DP-TV	0.975 (0.962–0.988)	0.975 (0.962–0.988)	1.000 (1.000–1.000)	0.975 (0.975–0.975)	Measure the blood flow velocity of various valves and blood vessels
DP-AO	0.946 (0.929–0.964)	0.928 (0.908–0.947)	1.000 (1.000–1.000)	0.937 (0.937–0.937)	
DP-MV	0.972 (0.958–0.985)	0.990 (0.982–0.998)	1.000 (1.000–1.000)	0.981 (0.981–0.981)	
DP-PV	0.761 (0.730–0.792)	0.730 (0.698–0.761)	0.999 (0.998–0.999)	0.745 (0.745–0.745)	
DP-DAO	0.921 (0.896–0.947)	0.862 (0.830–0.894)	1.000 (1.000–1.000)	0.891 (0.891–0.891)	
DP-OTHER	0.815 (0.796–0.835)	0.862 (0.845–0.880)	0.998 (0.997–0.998)	0.838 (0.838–0.838)	
Other	0.613 (0.604–0.622)	0.616 (0.607–0.626)	0.960 (0.959–0.961)	0.615 (0.615–0.615)	/
Total	0.865	0.848	0.994	0.853	/

*ASD, atrial septal defect; VSD, ventricular septal defect; PDA, patent ductus arteriosus; PS, pulmonary stenosis; AS, aortic stenosis; TOF, tetralogy of fallot; DORV, double outlet of right ventricle; CAVC, complete atrioventricular septal defect; COA, coarctation of aorta*.

**Figure 3:**
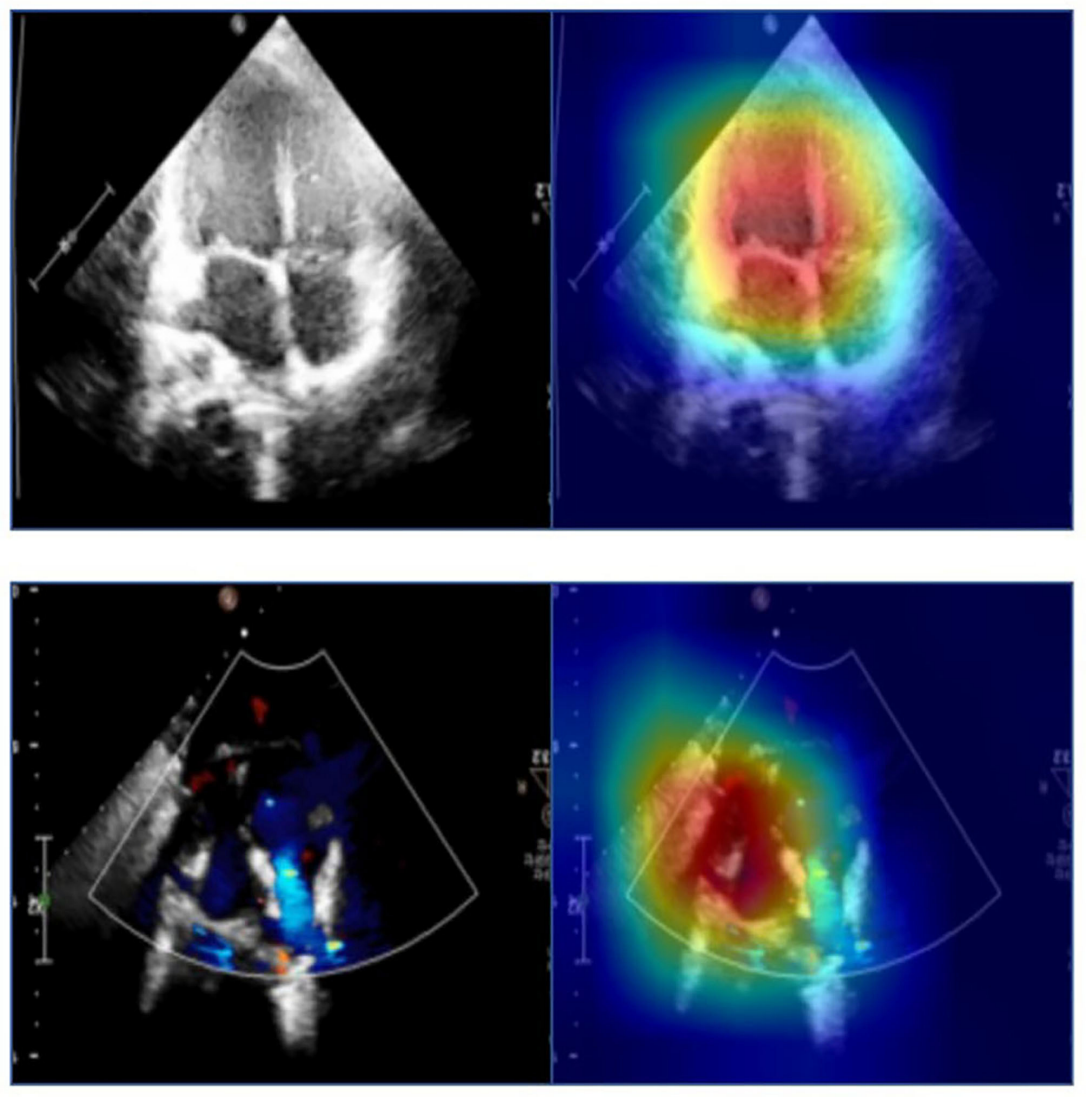
The activation maps of the apical four-chamber view and the subcostal sagittal view of the atrium septum. Different colors in the activation map represent different weights in model prediction. The red part has a higher weight and the blue part has a lower weight.

**Figure 4:**
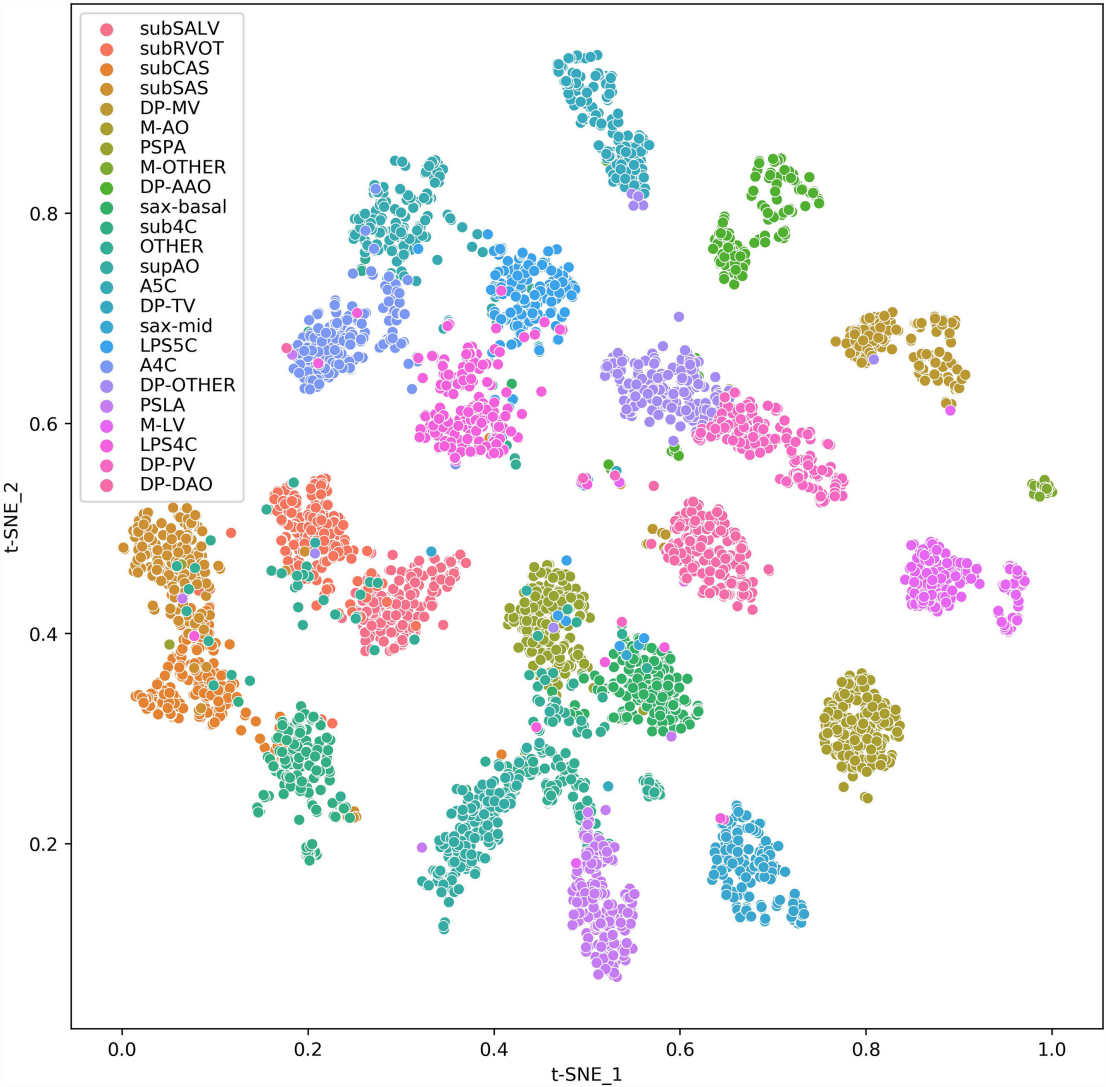
t-SNE visualization of CNN feature clusters for 24 echocardiographic views. Different views are represented with colored clusters and labels. The images are sampled from the test set data and 256 samples were randomly sampled for each view. For views whose total number are <256, all samples are applied.

**Figure 5:**
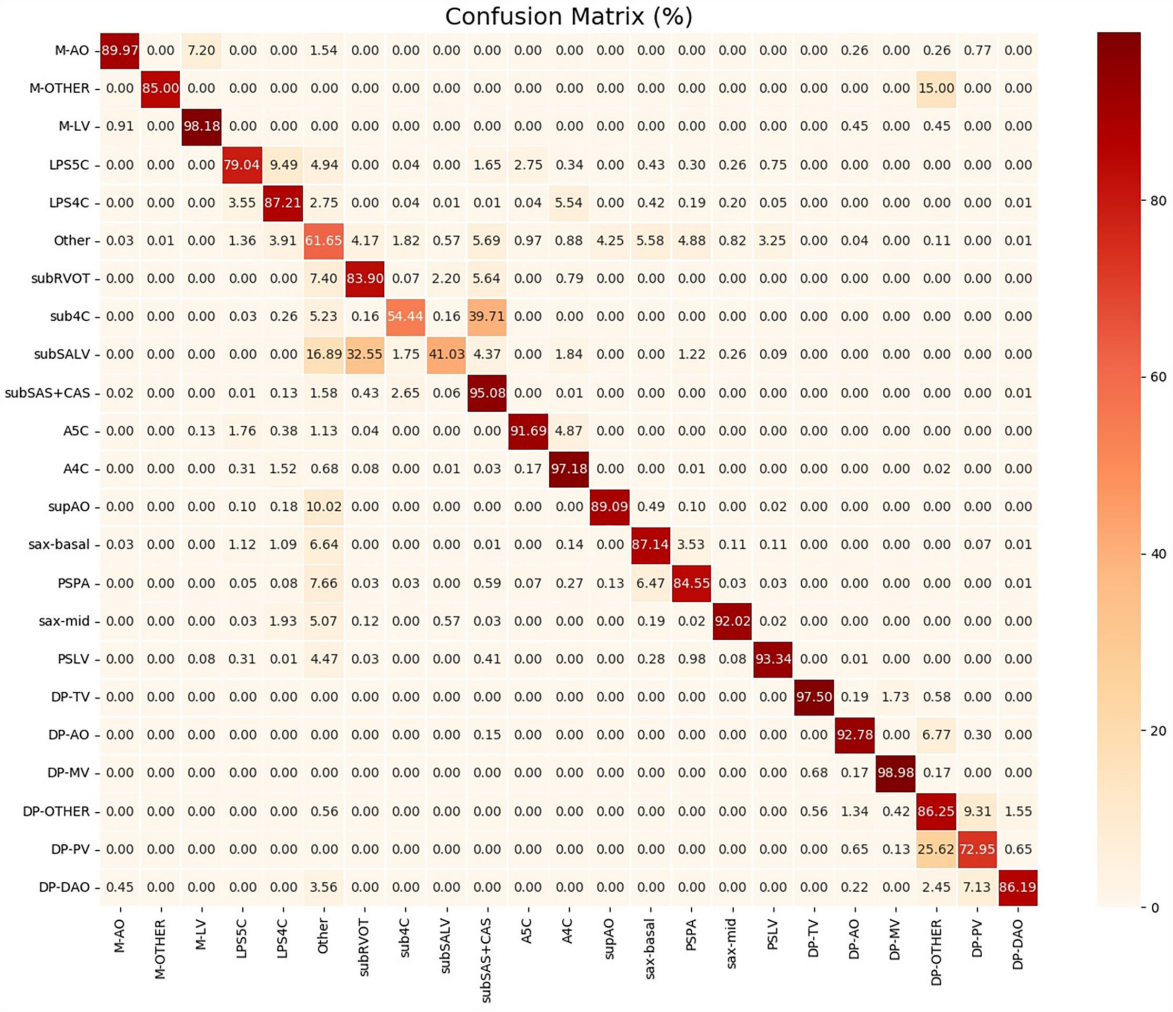
The confusion matrix between different echocardiographic views.

To help clinicians better understand the decision-making mechanism behind the model, the activation map can be visualized. As shown in Figure 3, the activation map reveals the attention distribution of the network, where different colors represent the weights of different pixels in the model's prediction process. The weight of the red part is larger, and the weight of the blue part is smaller. The t-distributed stochastic neighbor embedding (t-SNE) scatter plot in Figure 4 shows how our model clusters different views, where each point represents an image of a view in the test data, and its color represents its true view category. Each point in the scatter plot is a two-dimensional projection of the 512-dimensional feature vector generated by the last hidden layer of the model. We observe that most views can be separated well, and only a few clusters are mixed, which means our model can perform generally well on most views. The confusion matrix shown in Figure 5 also demonstrates that the proposed model performs well on most echocardiographic views. There are some confusion between subRVOT and subSALV, sub4C and subCAS+subSAS, LPS4C and LPS5C, and sax-basal and PSPA.

To further evaluate our model, we compared our model with ResNet-34 (the baseline student model), ResNeSt-200 (the teacher model), and two other widely used deep learning models, ResNeXt-50 (24) and DenseNet-161 (25). As shown in Table 4, our model and the baseline ResNet-34 model have the smallest parameters of 21.3 M. The parameters of the ResNeXt-50 model, Densenet-161 model, and ResNeSt-200 model are 23.0 M, 26.5 M, and 68.2 M, respectively. Our model achieved the highest sensitivity of 0.865 and specificity of 0.994, and the second best F1 score of 0.853, slightly lower than the highest F1 score of 0.854 of the teacher model. The precision rates of our model and the teacher model are 0.848 and 0.856, respectively, and the highest precision is 0.865 of DenseNet-161. With a comparable F1 score, the teacher network ResNeSt-200 is almost three times that of our model. Therefore, we can conclude that we achieved the best overall performance through knowledge distillation using the smallest model. In addition, an ablation study on pre-training using image-net was also carried out. Experimental results show that there are significant differences in precision (0.848 vs. 0.833), sensitivity (0.865 vs. 0.856), specificity (0.994 vs. 0.993), and F1 score (0.853 vs. 0.841) with and without pre-training, as shown in Table 4.

**Table 4:** Performance comparison.

**Model**	**Precision**	**Sensitivity**	**Specificity**	**F1 score**	**Parameters**
ResNet-34 (baseline/student)	0.812	0.845	0.992	0.820	**21.3M**
ResNeXt-50	0.817	0.830	0.992	0.822	23.0M
Densenet-161	**0.865**	0.826	0.993	0.838	26.5M
ResNeSt-200 (teacher)	0.856	0.856	**0.994**	**0.854**	68.2M
Our model (w/o weight pre-training)	0.833	0.856	0.993	0.841	**21.3M**
Our model^*^ (w/ weight pre-training)	0.848	**0.865**	**0.994**	0.853	**21.3M**

*Bold value indicates the best performance in the corresponding criteria*.

* *indicates the proposed method*.

## Discussion

In our study, we established a 24-category classification model to accurately and efficiently identify 23 commonly used standard echocardiographic views in the diagnosis of CHDs in children by using an effective deep learning-based neural network method through knowledge distillation. The most common CHDs in clinics include ASD, VSD, PS, PDA, TOF, and so on. One of the future work that we will conduct in the near future is to investigate how well the proposed model performs for specific congenital heart diseases (such as ASD).

Most of the ultrasound examinations for CHDs are the process of dynamic scanning where images acquired are mostly non-standard views and the image views are unevenly distributed. The images used in our study are all retrieved from the historical real examination database which is closer to the routine diagnosis of CHDs. This requires a trade-off between the complexity and accuracy of the model. The higher the model complexity, the higher the accuracy, but the operating efficiency will decrease. Therefore, we proposed a knowledge distillation method to maximize the accuracy of the network while keeping the complexity of the network unchanged. After training, the student network can be directly used to recognize the input ultrasonic image and achieve performance comparable to that of the teacher network without adding additional model complexity and time consumption.

Table 3 shows that F1 scores of the subCAS+SAS, A4C, A5C, LPS4C, sax-mid, sax-basal, PSLA, supAO, M-AO, M-LV, DP-MV, DP-TV, DP-AAO, and DP-DAO are all above or close to 0.90. Based on these views, common CHDs can be diagnosed, including ASD, VSD, PS, and TOF. Initially, subCAS and subSAS were treated as two separate views. However, due to the similarity of their image appearance, it is difficult to distinguish them. In addition, these two views are mainly used for the diagnosis of ASD and the scanning process is often between the two views, which makes the distinction more difficult. On the other hand, the confusion between the two views has no significant difference in the diagnosis of the disease. Therefore, we merged subCAS and subSAS into a new subCAS+SAS view.

As shown in Figure 5, the sub4C view was easily classified as subCAS, the subSALV view was easy to get misclassified as subRVOT, LPS5C view was easy to be misclassified as LPS4C and sax-basal. The main reason for these misclassifications was that these views were relatively close to each other. Moreover, in the scanning process, image slices were collected between these views, therefore it was more likely to cause misclassification. In addition, the training samples of the above views were also relatively small. Subcostal short-axis view of the left ventricle is primarily used to diagnose muscular VSDs, the information provided in this view can be supplemented by sax-mid. Parasternal view of the pulmonary artery was easily misclassified as sax-basal and “other” views. Through further analysis of misclassified images, we found that images that clearly showed the aortic root and pulmonary artery branches were easily misclassified. The low F1 score of the PV flow spectrum was caused by confusion with the blood flow spectrum of the pulmonary artery branches. In addition, the training data for the blood flow spectrum and the M-mode ultrasound was also small. In the future, we will add more training data to improve the performance of the proposed model.

## Conclusions

This study proposes an effective deep learning based method to identify echocardiographic views commonly used in the diagnosis of CHDs in children. Experimental results show that the proposed method can maximize the accuracy of the network while maintaining the complexity of the network. Compared with existing standard echocardiographic view recognition research, the images used in this study are all from the historical real examination database which are more realistic and authentic, providing foundation for subsequent AI identification of various types of CHDs with greater clinical significance.

## Data Availability Statement

The raw data supporting the conclusions of this article will be made available by the authors, without undue reservation.

## Ethics Statement

The studies involving human participants were reviewed and approved by the Medical Ethics Committee of Shanghai Children Medical Center. Written informed consent from the participants' legal guardian/next of kin was not required to participate in this study in accordance with the national legislation and the institutional requirements.

## Author Contributions

LW and XL analyzed the data and draft the manuscript draft. WH and LC reanalyzed the data. KG, QS, and YY did the statistical analysis. YZ, BD, and LZ conceived and designed the study, edited and reviewed the manuscript draft. All authors revised, read, and approval the final version of the manuscript.

## Funding

This work was supported by the ZheJiang Provincial Key Research Development Program (No. 2020C03073), the National Natural Science Foundation of China (No. 82001825), the Health and Family Planning Commission of China (No. 1132 GDEK201706), the Science and Technology Innovation-Biomedical Supporting Program of Shanghai Science and Technology Committee (No. 19441904400), and Program for artificial intelligence innovation and development of Shanghai Municipal Commission of Economy and Information, and the Biomedical and Engineering (Science) Interdisciplinary Study Fund of Shanghai Jiaotong University (No. YG2019QNB03).

## Conflict of Interest

XL, KG, QS, and YY were employed by Deepwise Healthcare. The remaining authors declare that the research was conducted in the absence of any commercial or financial relationships that could be construed as a potential conflict of interest.

## Publisher's Note

All claims expressed in this article are solely those of the authors and do not necessarily represent those of their affiliated organizations, or those of the publisher, the editors and the reviewers. Any product that may be evaluated in this article, or claim that may be made by its manufacturer, is not guaranteed or endorsed by the publisher.
